# Pharmaco-Toxicological Effects of *Cachrys libanotis* Extract: Antioxidant, Antimicrobial, and Cytotoxic Activities in Human Cell Lines and Embryonic Models

**DOI:** 10.3390/antiox14070810

**Published:** 2025-06-30

**Authors:** Mary Fucile, Ioana Gabriela Macasoi, Monica Negrea, Diana Obistioiu, Mariangela Marrelli, Ersilia Alexa, Cristina Dehelean, Giancarlo Statti, Filomena Conforti, Iulia Pinzaru

**Affiliations:** 1 Department of Pharmacy, Health and Nutritional Sciences, University of Calabria, 87036 Rende, Cosenza, Italy; mary.fucile@unical.it (M.F.); mariangela.marrelli@unical.it (M.M.); giancarlo.statti@unical.it (G.S.); 2 Faculty of Pharmacy, “Victor Babes” University of Medicine and Pharmacy, 2 Eftimie Murgu Square, 300041 Timisoara, Romania; macasoi.ioana@umft.ro (I.G.M.); cadehelean@umft.ro (C.D.); iuliapinzaru@umft.ro (I.P.); 3 Research Center for Pharmaco-Toxicological Evaluations, Faculty of Pharmacy, “Victor Babes” University of Medicine and Pharmacy, 2 Eftimie Murgu Square, 300041 Timisoara, Romania; 4 Faculty of Food Engineering, University of Life Sciences “King Mihai I”, Timisoara Aradului Street No.119, 300645 Timisoara, Romania; monicanegrea@usvt.ro (M.N.); dianaobistioiu@usvt.ro (D.O.); ersiliaalexa@usvt.ro (E.A.)

**Keywords:** coumarins, phytocomplexes, cytotoxic effect, antibacterial activity, irritant potential

## Abstract

We conducted a study to explore the potential of an enriched coumarin extract from *Cachrys libanotis* for the prevention and treatment of various diseases. The extract was prepared using pressurized cyclic solid–liquid extraction, and its safety profile was thoroughly evaluated using both cellular and embryonic models. Our main goal was to uncover a mixture of bioactive compounds that could offer therapeutic benefits. The following parameters were assessed: (i) extract composition; (ii) antioxidant activity; (iii) effects on cell viability and morphology; (iv) irritant potential (in ovo); and (v) antimicrobial activity against nine microbial strains. Chromatographic and spectrometric analyses confirmed that the main specialized metabolites in *C. libanotis* extract were furanocoumarins, with xanthotoxin, bergapten, and isopimpinellin identified as the three predominant constituents. Treatment with the *C. libanotis* extract did not induce significant alterations in the adherent human keratinocytes, with confluence and epithelial morphology comparable to control cells. Conversely, viable cells declined in the breast carcinoma cell line (MDA-MB-231). Moreover, the *C. libanotis* extract exhibited a promising antimicrobial activity against two Gram-negative pathogens, *Shigella flexneri* and *Salmonella typhimurium*.

## 1. Introduction

Plants are a major source of bioactive compounds with significant potential for the prevention and treatment of various diseases. Biomedical research today heavily investigates plant-derived secondary metabolites given their broad spectrum of biological activities, which encompass antimicrobial, antifungal, anticancer, and anti-inflammatory properties [1]. Bioactive constituents present in plant extracts, along with phytocomplexes (synergistic mixtures of secondary metabolites), are of particular interest for their potential applications in chemoprevention. In numerous disorders such as cancer, diabetes, cardiovascular diseases, and mutagenesis—caused by cellular damage or oxidative injury—antioxidants play a preventive role. Recognizing the critical challenge posed by cancer, the National Cancer Institute has prioritized the discovery of novel natural compounds and phytocomplexes, allocating substantial resources toward research in this area [2]. Infectious diseases represent another escalating global health threat, driven in large part by the rise of antibiotic-resistant microorganisms. Resistant bacterial infections are now considered a global emergency, and without effective countermeasures, The World Health Organization projects that antimicrobial resistance could lead to approximately 10 million deaths annually by 2050 [3]. This crisis has renewed interest in plant-based traditional medicine as a primary form of healthcare. Beyond demonstrating biological efficacy, it is essential to evaluate the safety profiles of plant-derived extracts through biocompatibility assessments and analyses of potential irritant effects [4].

Within this context, members of the *Cachrys* genus have emerged as promising candidates for the development of new therapeutic agents. Previous studies have demonstrated that *Cachrys* species possess photocytotoxic potential against human melanoma cells. Specifically, *C. pungens* exhibited strong photocytotoxic effects on A375 melanoma cells following UVA irradiation [5]. Similarly, *C. sicula* and *C. libanotis* showed photocytotoxic activity against a C32 human melanoma cell line. Several phytochemicals, including coumarins, have demonstrated photosensitizing properties, enhancing their anticancer effects. This is due to the high production of local reactive oxygen species (ROS) during the photochemical reaction, which drives cancer cells toward apoptosis and/or necrosis [6,7].

Investigations using different extraction methods suggested that pressurized cyclic solid–liquid extraction yields an improved phytochemical profile conducive to enhanced anticancer activity [8]. A variety of factors can influence the chemical composition of plants, including environmental conditions, as well as extraction and isolation procedures [9]. Although much research exists, there are fewer studies on breast cancer cell lines. However, the existing literature supports their promise. Our previous studies demonstrated that *Cachrys* species could serve as promising candidates for the discovery of new drugs with photocytotoxic potential [5]. Different extraction methods produced extracts with varying levels of activity, with the pressurized cyclic solid–liquid extraction technique yielding a superior phytochemical composition against human C32 melanoma cells irradiated with UVA light [8,10]. The antiproliferative effectiveness of novel bioconjugates incorporating 3-substituted coumarins and estradiol was examined in one study, which uncovered the potent antiproliferative activity of these compounds on noninvasive and invasive breast cancer cell lines (MDA-MB-231/ATCC and NCI/ADR-RES MDA-MB-435) [11]. Furthermore, Cui et al. (2019) [12] reported that three synthesized coumarins, originating from triphenylethylene (TCHs), showed anticancer effects by suppressing angiogenesis in breast cancer cell lines. The best-known property of furanocoumarins is phototoxicity. It is known that furanocoumarins form monoadducts with pyrimidine bases of DNA. Psoralen, bergapten, and xanthotoxin exhibit high cytotoxicity. Furthermore, their phototoxicity levels vary under UV influence. For disubstituted (isopimpinellin), geranylated (bergamottin), or angular furanocoumarins, phototoxicity is considerably reduced. Angular furanocoumarins either lack phototoxic properties or form only monoadducts, not interstrand bonds. Apoptosis induction occurs through various cell signaling systems and transcription factors, which are impacted by furanocoumarin structures [13]. For instance, nuclear factor kappa of activated B cells (NF-κB), linked to cancer development and inflammatory processes, is inhibited by furanocoumarins (e.g., psoralen [14]), thus suppressing subsequent negative processes. Phosphatidylinositol 3-kinase (PI3/Akt) typically inactivates caspases and other apoptotic enzymes. However, furanocoumarins, such as xanthotoxin [15], activate these enzymes by suppressing the PI3/Akt pathway. The p53 factor suppresses tumor development by disrupting the cell cycle and stimulating apoptosis, and furanocoumarins enhanced its expression (e.g., imperatorin [16]).

Some references focused on the antimicrobial activity of different extracts of *C. cristata* [10]. Methanol, ethyl-acetate, acetone, and water extracts derived from the aerial parts and fruits of *C. cristata* from the Serbian area were evaluated for their antimicrobial activity. The tested organisms included common human gastrointestinal pathogenic bacteria (*E. coli*, *P. aeruginosa*, *S. enteritidis*, *B. cereus*, *L. monocytogenes*, *S. aureus*) and the yeast *C. albicans*. Lower concentrations for the MIC, compared to those obtained by us, were reported for the methanolic extracts analyzed for *E. coli*, *B. cereus* (0.78 mg/mL), *S. aureus* (0.39 mg/mL), *C. albicans* (6.25 mg/mL), and *L. monocytogenes* (12.5 mg/mL); however, no bactericidal effect was observed at concentrations of 50.0 mg/mL.

Building on previous discoveries, we examined the toxicological profiles of an enriched coumarin extract from *Cachrys libanotis*, prepared via pressurized cyclic solid–liquid extraction. Specifically, the study assessed the following: (i) extract composition; (ii) antioxidant activity; (iii) effects on cell viability and morphology; and (iv) irritant potential (in ovo). Additionally, the antimicrobial activity of the extract against nine microbial strains was investigated.

## 2. Materials and Methods

### 2.1. Chemicals

In the present study, the following reagents were applied: specific culture media: Eagle’s Minimum Essential Medium (EMEM) and Dulbecco’s Modified Eagle’s Medium (DMEM), cell culture supplements—heat-inactivated fetal bovine serum (FBS), antibiotics (penicillin, streptomycin), phosphate-buffered saline (PBS), trypsin-EDTA solution, and an MTT assay determination kit, which were purchased from ATCC (American Type Culture Collection, Virginia, VA, USA), Thermo Fisher Scientific, Inc. (Waltham, MA, USA), and Sigma Aldrich, Merck Kga Group (Darmstadt, Germany), respectively. The reagents used in the experiments were of analytical purity and approved for cell culture use. All solvents used were reagent-grade and were purchased from VWR International s.r.l. (Milan, Italy).

### 2.2. Plant Collection and Storage

Aerial parts from *C. libanotis* were collected in Calabria (Italy) in August 2023 during the flowering period and identified by Filomena Conforti. A voucher specimen has been deposited at the Mediterranean Etnobotanical Conservatory, Sersale (Catanzaro), in the Apiaceae section (numbers 18). Samples underwent room-temperature drying for approximately two weeks until the leaves and flowers were easily friable. The resulting dried material was then pulverized into a fine powder with a blender and stored in airtight, dark bottles prior to extraction.

### 2.3. Extraction

Dried samples were extracted with EtOH 70% through pressurized cyclic solid–liquid extraction (plant-to-solvent ratio 1:10 g/mL). This solid–liquid dynamic method is particularly effective, as it allows for faster extraction than conventional maceration techniques. The extract obtained was filtered through Whatman no. 1 filter paper and dried under vacuum to determine the weight. The plant material underwent two extraction cycles to ensure complete recovery. After determining the mass of the obtained extracts (yield 13%), each was redissolved in 70% EtOH to a 100 mg/mL stock solution. These solutions were used to create different working concentrations for quantitative phytochemical analysis and biological assays. All samples were stored at 4 °C until analysis.

### 2.4. Compositional Analysis of the Extract

The coumarin content in the hydroalcoholic extract was determined via gas chromatography–mass spectrometry (GC-MS), adapting the analytical procedures described in Marrelli et al. (2021) [5]. Phytochemical profiling was conducted on a Hewlett-Packard 6890 gas chromatograph featuring an SE-30 capillary column (100% dimethylpolysiloxane, 30 m × 0.25 mm ID × 0.25 µm film) coupled to a Hewlett-Packard 5973 selective mass detector. Compound identification relied on comparing GC retention factors against authentic standards and matching mass spectra with entries from the Wiley library.

### 2.5. Bovine Brain Peroxidation Assay

To assess antioxidant activity, hydroalcoholic extracts (from 5 µg/mL to 1000 µg/mL) were tested against liposomes prepared from bovine brain extract (5 mg/mL) in phosphate-buffered saline. This method relied on the formation of malondialdehyde (MDA), a byproduct generated in small amounts during the peroxidation of most membrane systems. Malondialdehyde (MDA) was reacted with thiobarbituric acid (TBA) to generate a colored complex, with its absorbance then quantified at 532 nm. The absorbance values of liposomes alone and of the extract alone were also considered in the analysis. Propyl gallate served as a positive control in the assay.

### 2.6. Cell Lines and Cell Culture Growth

To conduct the present experiment, the following cell lines were used: a healthy cell line—HaCaT, human immortalized keratinocytes (acquired from CLS Cell Lines Service GmbH, Eppelheim, Germany)—and a tumor cell line—MDA-MB-231, human breast adenocarcinoma cells, triple-negative phenotype (obtained from ATCC—American Type Culture Collection, as a frozen vial). The cells (HaCaT and MDA-MB-231) were grown according to the manufacturer’s protocol in specific culture media, as follows: DMEM (ATCC 30-2002—for HaCaT) and EMEM (ATCC 30-2003—for MDA-MB-231), which were supplemented with FBS (final concentration 10%) and a mixture of antibiotics (penicillin/streptomycin—final concentration 1%). During the experiment, the cells were kept in a humidified CO_2_ incubator in standard conditions: 37 °C and 5% CO_2_. The number of cells/well was counted using a Countess II Automated Cell Counter (Thermo Fisher Scientific, Inc., Waltham, MA, USA) in the presence of Trypan blue.

### 2.7. Cell Morphology Assessment

The impact of the test extract (at concentrations between 50 and 150 µg/mL) on cell morphology and confluence was monitored microscopically using Cytation 1 (BioTek Instruments Inc., Winooski, VT, USA). Cells were observed under bright-field illumination, and images were captured at the conclusion of the 24 h treatment period. The acquired images were analyzed using the Gen5™ Microplate Data Collection and Analysis Software Version 3.14 (BioTek Instruments Inc., Winooski, VT, USA).

### 2.8. Cell Viability and Schratch Assay

To assess the effect of the test plant extracts on cell viability, the MTT (3-(4,5-dimethylthiazol-2-yl)-2,5-diphenyltetrazolium bromide) method was performed. The protocol applied was based on previous studies and the manufacturer’s specifications and adapted to our laboratory conditions. In brief, the MDA-MB-231 and HaCaT cells were cultured in 96-well plates (10^4^ cells/200 µL/well) and stimulated with different concentrations of test plant extracts (50–100 µg/mL) for 48 h. The dilution of the plant extract (lyophilized) was carried out using the culture media. After 24 h, the old culture media was replaced with 100 µL of fresh media and 10 µL of MTT reagent/well, followed by a 3 h incubation at 37 °C. After the incubation step, the solubilization buffer (100 µL/well) was added, and the plates were maintained at room temperature for 30 min and protected from light. The absorbance values were measured at two wavelengths (570 and 630 nm) using Cytation 5 (BioTek Instruments Inc., Winooski, VT, USA). To investigate the impact of *C. libanotis* extract (Cex) on the migratory behavior of MDA-MB-231 cells, a wound healing assay was conducted. Cells were seeded into 24-well plates and allowed to grow until a confluent monolayer formed. A standardized scratch was introduced using the AutoScratch™ Wound Making Tool (BioTek^®^ Instruments Inc., Winooski, VT, USA) in accordance with the manufacturer’s guidelines. Immediately after wounding (T0), images were taken to document the initial scratch area using the Cytation 1 imaging system. Cells were then exposed to three concentrations of the extract (50, 100, and 150 μg/mL) for a period of 24 h. These concentrations were selected based on prior viability results, which showed a graded response and allowed for the assessment of migration without excessive cytotoxic interference. After 24 h (T24), images were captured again, and the extent of wound closure was analyzed using the Gen5™ software. The migration rate was calculated according to a previously established method [17].

### 2.9. The HET-CAM Assay: A Chorioallantoic Membrane-Based Test Using Hen’s Eggs

The HET-CAM method was utilized to assess the extract’s toxicity. Chicken eggs were prepared as follows: Locally purchased eggs were washed and disinfected with 70% ethanol. After noting the incubation date, the eggs were placed in an incubator at a constant 37 °C with consistent humidity. On the fourth day of incubation, a perforation was made in the eggshell to extract approximately 7–8 mL of fluid. This allowed for the detachment of the inner shell membrane, improving the visualization of blood vessels. The following day (fifth day of incubation), a window was cut into the egg’s upper surface, which was then covered with adhesive tape, and the eggs were returned to the incubator until the experiment’s start. The HET-CAM test itself was performed on the ninth day of incubation. A 1% sodium dodecyl sulfate sample served as the positive control, while water was used as a negative control. Samples (100 µg/mL) and both controls, in a 600 µL volume, were applied to the chorioallantoic membrane (CAM) to ensure complete coverage. Vascular changes were monitored for 5 min. The evaluation of potential irritating effects and biocompatibility was carried out by photographing the membrane before (T0) and 5 min after (T5) application, analyzing for hemorrhage (H), coagulation (I), and vascular lysis (IT). Images and microscopic evaluations were conducted using an Axio CAM 105 color, Zeiss, a Discovery 8 Stereomicroscope, and the Image J software v 1.50e. The irritation score (IS) was calculated to quantify vascular effects [18]. Based on the IS value, substances were classified into three categories: non-irritating (IS = 0–0.9), irritating (IS = 1–8.9), and severely irritating (IS = 9–21) [19].

### 2.10. Antimicrobial Activity Test

To test the antimicrobial activity, an alcoholic extract (70%) of *C. libanotis* was prepared, and then different quantities were spotted into 96-well plates to reach the 3.5%, 5%, 10%, and 15% concentrations selected by our study. The samples were tested against different strains. Gram-positive: *Streptococcus pyogenes* (ATCC 19615), *Staphylococcus aureus* (ATCC 25923), *Listeria monocytogenes* (ATCC 19114), *Bacillus cereus* (ATCC 10876); Gram-negative: *Shigella flexneri* (ATCC 12022), *Escherichia coli* (ATCC 25922), and *Salmonella typhimurium* (ATCC 14028) and yeasts represented by *Candida albicans* (ATCC 10231) and *Candida parapsilopsis* (ATCC 22019).

Initially, an attempt was made to establish the minimum inhibitory concentration (MIC) for each extract. The MIC is defined as the lowest compound concentration that yields no visible microorganism growth. The method of MIC determination based on the microbial mass loss by measurement of OD by spectrophotometry according to ISO 20776–1:2019 was described in our previous research [20].

#### 2.10.1. Bacterial Culture

To prepare the bacterial culture, a 10^−2^ dilution of the fresh culture and an inoculum equivalent to a 0.5 McFarland (1.5 × 10^8^ UFC × mL), detected using a McFarland Densitometer (Grand-Bio, Cambridge, England), were used.

To start the experiment, bacterial strains were first revived through overnight growth in brain heart infusion (BHI) broth (Oxoid, CM1135) at 37 °C. Subsequently, these strains were transferred to BHI Agar (Oxoid, CM1136) and incubated for an additional 24 h at 37 °C. For the assay, 100 µL aliquots of the prepared microbial suspensions were dispensed into individual wells of 96-well microdilution plates. The various extracts under investigation were then added to these wells, achieving final concentrations of 3.5 mg/mL, 7 mg/mL, 10 mg/mL, and 15 mg/mL. The plates were covered and allowed to incubate for 24 h at 37 °C. Following incubation, the optical density (OD) of each well was determined at 540 nm using an ELISA reader (BIORAD PR 1100, Hercules, CA, USA). Each sample was analyzed in triplicate to ensure reproducibility. Gentamicin (10 μg/mL) (Oxoid Ltd., Thermo Fisher Scientific, Basingstoke, UK) was used as a positive control for antibacterial activity, while the BHI broth with bacterial suspension served as the negative control. Negative controls consisted of the bacterial strain suspension without extract, and the values obtained were used as the control in the interpretation

For interpretation of the results, the bacterial inhibition percentage (BIP) was calculated according to the formula:

$$\mathrm{BIP}=100-\frac{OD_{\mathrm{sample}}}{OD_{\mathrm{negative}\;\mathrm{control}}}\times 100(\%)$$

where

OD_sample_—optical density for extract in the presence of the selected bacteria;

OD_negative control_—optical density for the selected bacteria in BHI.

#### 2.10.2. Fungal Culture

Fungal cultures were prepared following the same protocol as the bacterial cultures, with two key differences: fungal strains were revived for 48 h at 37 °C, and the cultures were diluted to an OD of 0.5 McFarland standard using BHI broth. Extracts were tested at the same concentrations as in the bacterial trials. For each test, 100 µL of fungal suspension was placed into wells of a 96-well microdilution plate. The plates were covered and incubated for 48 h at 37 °C, after which the OD was measured at 540 nm. All samples were tested in triplicate. Fluconazole (25 μg/mL) (Oxoid Ltd., Thermo Fisher Scientific, Basingstoke, UK) was used as a positive control for antifungal activity, and BHI broth with fungal suspension served as the negative control. Negative controls consisted of the fungal strain suspension without extract, and the OD values obtained were used as the control in the interpretation.

For interpreting the results, the mycelium growth percentage (MIP) was calculated according to the following formula:

$$\mathrm{MIP}=100-\frac{OD_{\mathrm{sample}}}{OD_{\mathrm{negative}\;\mathrm{control}}}\times 100(\%)$$

where

OD_sample_—optical density of extracts in the presence of the selected fungi;

OD_negative control_—optical density for the selected fungi in BHI.

### 2.11. Statistical Analysis

The presented results represent the average of the obtained values, along with their standard deviation (SD). All calculations were performed using the statistical software IBM SPSS 22. Antimicrobial activity rates, figures, and dose–effect relationships were performed using Microsoft Excel 365 (Version 2208, Washington, DC, USA).

## 3. Results

### 3.1. Coumarin Composition of the Hydroalcoholic Extract

**Figure 1:**
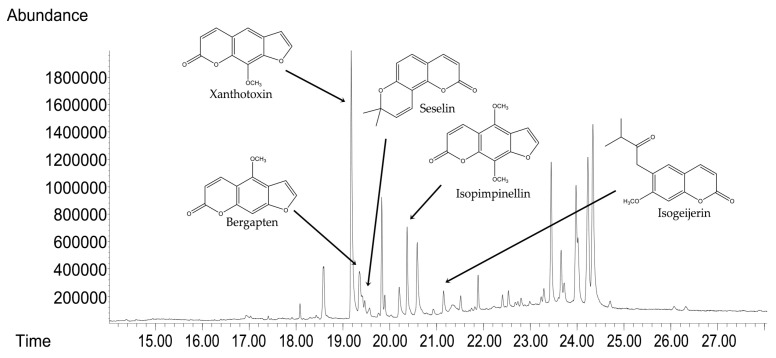
GC-MS chromatograms of *C. libanotis* extract.

GC-MS analyses were used to determine the compositional profile of the prepared hydroalcoholic extract from the aerial parts of *C. libanotis*. Compounds were identified according to their retention times and mass spectra (Supporting Material). As expected, ref. [5] the extract contained a high concentration of coumarins. Based on chromatographic and spectrometric data, furanocoumarins were confirmed as the predominant specialized metabolites, with xanthotoxin (5 mg/mL), bergapten (0.60 mg/mL), and isopimpinellin (0.43 mg/mL) as the three dominant constituents. The extract developed for the present investigation was also found to contain the pyranocoumarin seselin and the coumarin isogeijerin (Figure 1).

### 3.2. Antioxidant Activity

**Figure 2:**
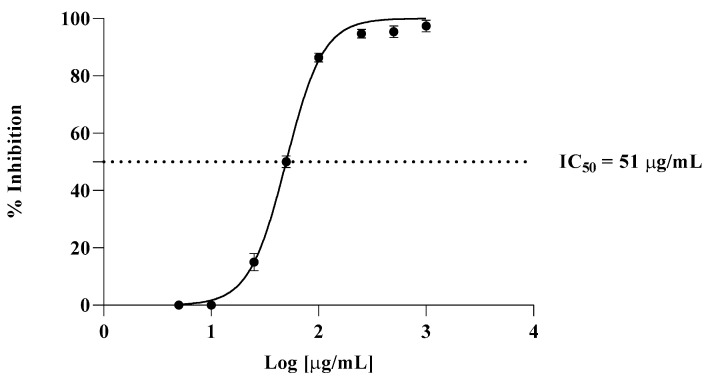
Inhibition of lipid peroxidation by *C. libanotis* extract.

The TBA test was employed to evaluate in vitro antioxidant activity, in which liposomes were prepared from bovine brain. The effects of various amounts of *Cachrys* extract (from 5 µg/mL to 1000 µg/mL) on lipid peroxidation of liposomes are presented in Figure 2. An IC_50_ of 7 μg/mL was observed for propyl gallate, used here as a positive control.

### 3.3. Cytotoxicity Assessment

A healthy human keratinocyte cell line (HaCaT) and a breast carcinoma cell line (MDA-MB-231) were used to evaluate the potential cytotoxic effects of the *Cachrys* extract (Cex).

**Figure 3:**
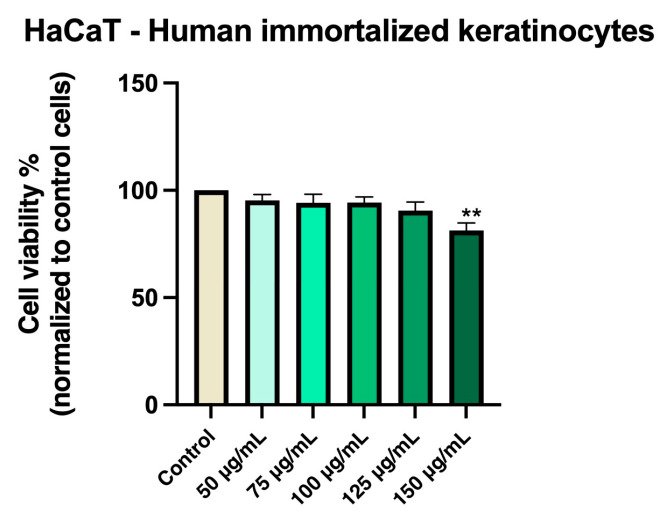
In vitro evaluation of Cex (50, 75, 100, 125, and 150 μg/mL) on HaCaT cell viability at 48 h post-treatment using the MTT assay. Results are expressed as the percentage (%) of cell viability normalized to the control (untreated cells). Data represents the mean ± standard deviation (SD) of three independent experiments performed in triplicate. Statistical differences compared to control cells were determined by one-way ANOVA followed by Dunnett’s multiple comparison post-hoc test (** *p* < 0.01).

HaCaT cells maintained high viability (approximately 95%) when treated with Cex at concentrations between 50 and 100 µg/mL. A modest reduction in viability was observed at 125 and 150 µg/mL, though cell viability remained above 81% (Figure 3).

**Figure 4:**
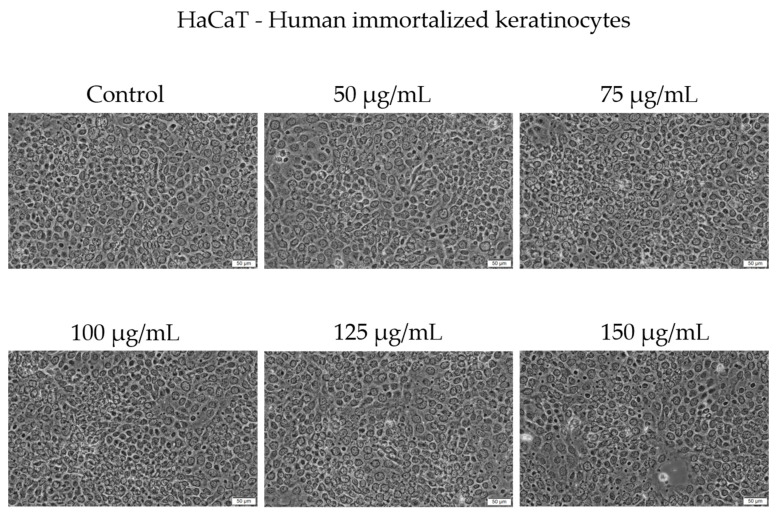
The morphological changes in HaCaT cells after 48 h of treatment with Cex (50, 75, 100, 125, and 150 µg/mL). Scale bar = 50 μm.

Morphologically, HaCaT cells preserved a typical epithelial phenotype and conflu-ence across concentrations up to 125 µg/mL. At 150 µg/mL, a slight decrease in cell con-fluence was noticed; however, no major morphological alterations were observed under bright-field microscopy (Figure 4).

**Figure 5:**
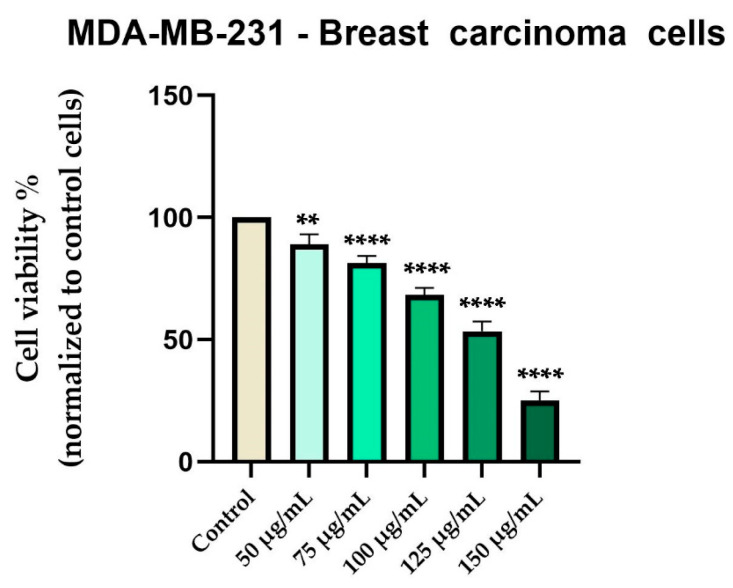
In vitro evaluation of Cex (50, 75, 100, 125, and 150 μg/mL) on the viability of MDA-MB-231 cells at 48 hours post-treatment using the MTT assay. Results are expressed as the percentage (%) of cell viability normalized to the control (untreated cells). Data represents the mean ± standard deviation (SD) of three independent experiments performed in triplicate. Statistical differences compared to control cells were determined by one-way ANOVA analysis followed by Dunnett’s multiple comparisons post-hoc test (** *p* < 0.01 and **** *p* < 0.0001).

In contrast, Cex reduced the viability of MDA-MB-231 breast carcinoma cells in a concentration-dependent manner, with a gradual decline beginning at 50 µg/mL (89%) and reaching approximately 25% at 150 µg/mL (Figure 5).

**Figure 6:**
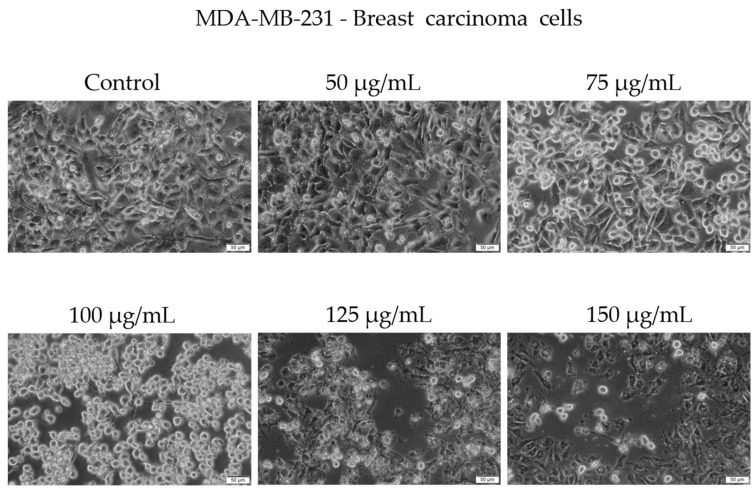
Representative images showing the morphological appearance of MDA-MB-231 cells after 48 h of treatment with *Cachrys libanotis* extract (Cex) at concentrations of 50, 75, 100, 125, and 150 μg/mL. Notably, more prominent morphological changes (e.g., cell rounding, partial detachment) were observed at intermediate concentrations (75 and 100 μg/mL), whereas at higher concentrations (125 and 150 μg/mL), fewer adherent cells remained due to extensive cell death and detachment, as also reflected in the viability data. Scale bar = 50 μm.

Morphological examination showed early signs of cytotoxicity at 50 µg/mL, such as partial cell rounding and detachment. At higher concentrations, particularly 125 and 150 µg/mL, the cells displayed more pronounced features consistent with loss of viability, including reduced confluence, rounding, loss of adhesion, and presence of floating cells and cellular debris (Figure 6).

It is important to note that while some adherent cells were still visible at 150 µg/mL, the overall cell population was markedly reduced compared to the control, and surviving cells exhibited an altered phenotype. We acknowledge that the visual evidence at this concentration, although supportive of a cytotoxic effect, may not be fully conclusive in isolation. Our interpretation is therefore based on a combination of viability assay data and qualitative morphological trends observed across replicates.

### 3.4. Cell Migration

**Figure 7:**
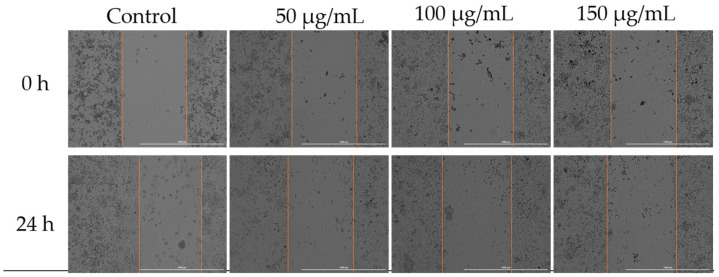
Effect of Cex (50, 100, and 150 µg/mL) on the migratory capacity of MDA-MB-231 cells following 24 h of treatment. Scratch widths were photographed at the 0 and 24 h marks following exposure. All scale bars denote 1000 µm.

To assess how Cex affected the migratory capacity of MDA-MB-231 cells, a key characteristic of cancer cells, the scratch assay method was employed. Three concentrations were selected for analysis: 50, 100, and 150 µg/mL. According to the results obtained, the highest migration rate was observed in the untreated control cells. Treatment with 50 µg/mL of Cex resulted in slight inhibition of cell migration; however, increasing the concentration to 125 µg/mL, and particularly 150 µg/mL, led to a significant inhibition of migration (Figure 7).

### 3.5. In Ovo Assessment of Plant Extract’s Biocompatibility and Irritant Potential

The HET-CAM assay was used to evaluate the extract’s biocompatibility and its potential as an irritant. To better understand the sample’s toxicological profile, a negative control (H_2_O) and a positive control (1% SDS) were included. Table 1 presents the irritation scores (IS) along with the bleeding (tH), lysis (tL), and coagulation (tC) times recorded. The *C. libanotis* extract exhibited a low IS value (0.78) compared to the positive control (19.41), indicating minimal irritant potential.

**Figure 8:**
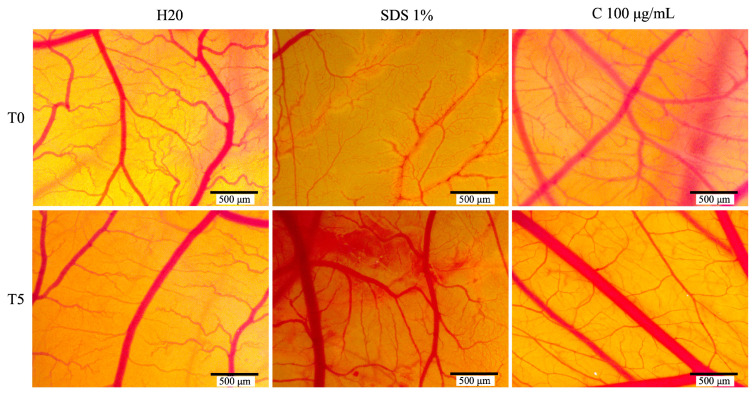
The stereomicroscope images depict the CAMs treated with controls (H_2_O, acting as the negative control, and sodium dodecyl sulfate, SDS, as the positive control) and Cex.

As expected, the positive control (1% SDS) yielded the highest irritation score of 19.41, with the negative control (H_2_O) showing the lowest score. The *C. libanotis* extract exhibited a similarly low irritation score of 0.78. This result suggests that the extract does not induce irritant effects on the chorioallantoic membrane and demonstrates a favorable biocompatibility profile. No major alterations in the vascular plexus were observed following treatment with the extract (Figure 8).

### 3.6. Antimicrobial Activity

**Figure 9:**
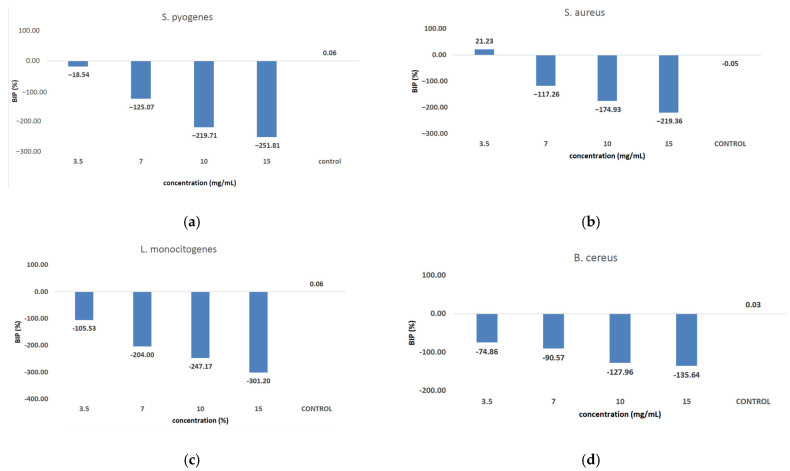

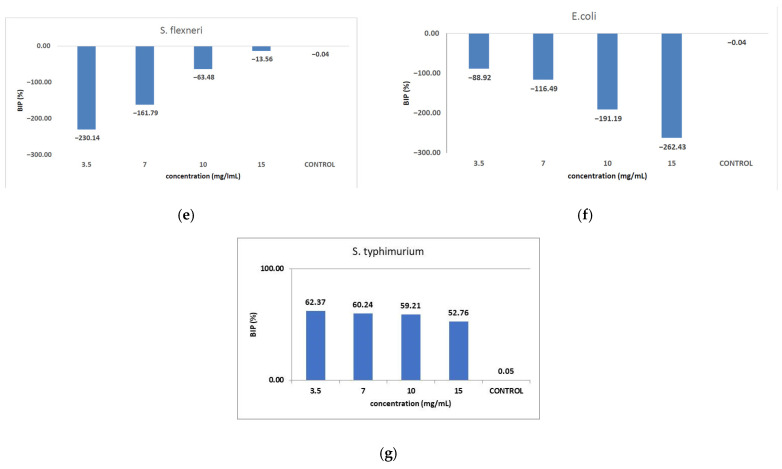
Inhibition potential of the extract against Gram-positive and Gram-negative bacteria. BIP values (%) of analyzed extracts against: (**a**) *S. pyogenes*; (**b**) *S. aureus*; (**c**) *L. monocytogenes*; (**d**) *B. cereus*; (**e**) *S. flexeneri*; (**f**) *E. coli*; (**g**) *S. typhimurium*.

Figure 9 shows the inhibition potential of *C. libanotis* extract, expressed as BIP (%), for all bacterial strains tested.

The extract led to an unexpected stimulation of *S. pyogenes* growth (Figure 9a), where the level of stimulation was negatively correlated with the increase in concentration. Starting with a concentration of 3.5 mg/mL, the BIP% was 18.54%, increasing to 251.81% at the highest concentration, with no discernible MIC value. In terms of its effectiveness against *S. aureus* (Figure 9b), the extract exhibited a bacterial inhibition percentage (BIP) of 21.23% at a concentration of 3.5 mg/mL. On the contrary, at higher concentrations (7, 10, and 15 mg/mL), an unexpected bacterial growth was noted, resulting in negative BIP values, demonstrating a direct link to the extract concentration. Regarding the inhibition of *L. monocytogenes* (Figure 9c), a proportional increase in bacterial growth corresponding to higher extract concentrations was noted. The BIP values ranged from −105.53% at the lowest concentration tested (3.5 mg/mL) to −301.2% at 15 mg/mL, signifying a notable increase in bacterial growth relative to the control. Similarly, the extract did not inhibit *B. cereus* (Figure 9d), as evidenced by negative BIP values between −74.86% and −135.64%, indicating a stimulatory effect on bacterial growth. Among the Gram-positive strains analyzed, the extract exhibited a stimulatory effect on bacterial growth, showing an increase of up to 219.36% compared to the control. This growth enhancement, observed across all Gram-positive strains, suggests the presence of chemical compounds within the extract that facilitate bacterial proliferation. The stimulating effect correlates with the increase in the concentration of the extract. Inhibition tests on *S. flexneri* (Figure 9e) indicated that higher applied concentrations resulted in greater growth inhibition, peaking at a concentration of 15 mg/mL, comparable to the negative control. The inhibition percentage at 3.5 mg/mL was −230.14%, which improved to −13.56% at 15 mg/mL. This trend suggests that the minimum inhibitory concentration (MIC) may be approximately 20 mg/mL. Further research is necessary to ascertain the lowest concentration with bactericidal or bacteriostatic effects. *E. coli* was identified as one of the least sensitive strains (Figure 9f), with BIP% values ranging from −88.92% to −262.43%, and no MIC was detected. In the analysis against *S. typhimurium*, a positive BIP (%) was recorded, ranging from 62.37% at the lowest concentration of 3.5 mg/mL to 52.76% at 15 mg/mL (Figure 9g). Despite positive inhibition values, these decreased with higher extract concentrations, suggesting that certain minor compounds may enhance bacterial growth.

**Figure 10:**
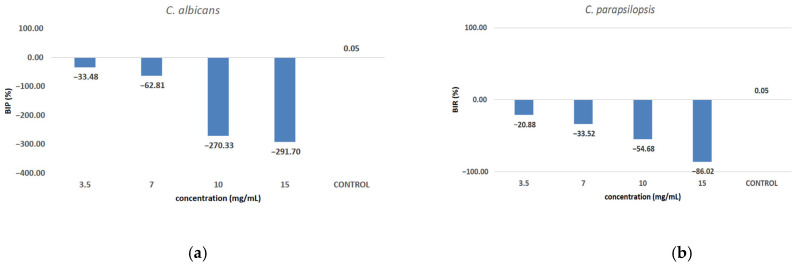
The inhibition potential of extract against fungi: (**a**) the BIP (%) of the analyzed extract against *C. albicans*, (**b**) the BIP (%) of the analyzed extract against *C. parapsilopsis*.

Against *C. albicans* (Figure 10a), the extract showed a strain-boosting effect, with BIP% values ranging from −33.48% to −291.70%. Regarding *C. parapsilopsis*, the activity was quite similar to *C. albicans*, the difference being given only by different values of BIP%, with all the extracts showing a better activity against *C. albicans* than against *C. parapsilopsis* (Figure 10b).

**Figure 11:**
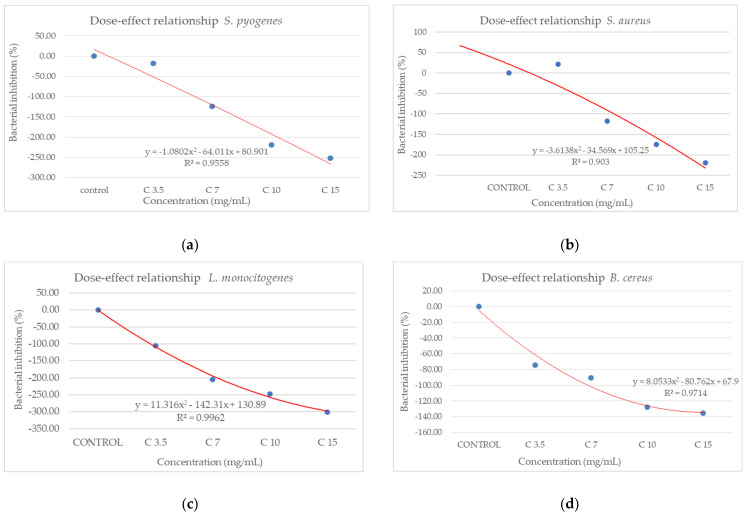

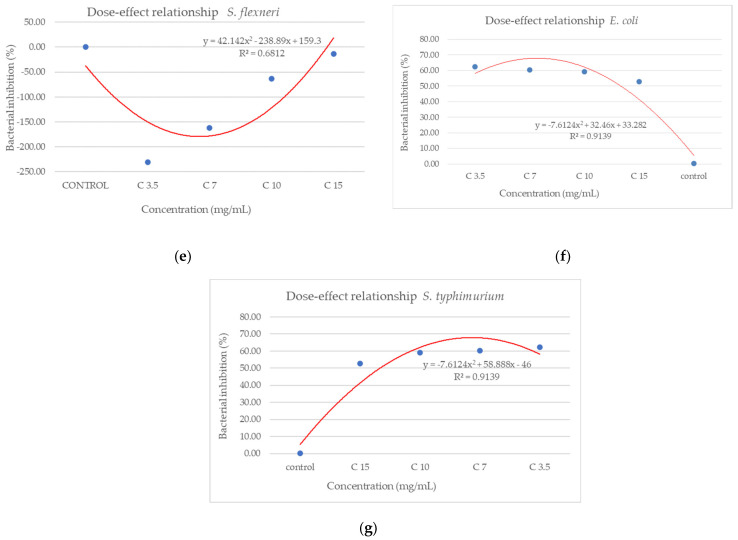
Dose–effect curves of the analyzed extract as a function of concentration (mg/mL) against (**a**) *S. pyogenes*; (**b**) *S. aureus*; (**c**) *L. monocytogenes*; (**d**) *B. cereus*; (**e**) *S. flexeneri*; (**f**) *E. coli*; (**g**) *S. typhimurium*.

**Figure 12:**
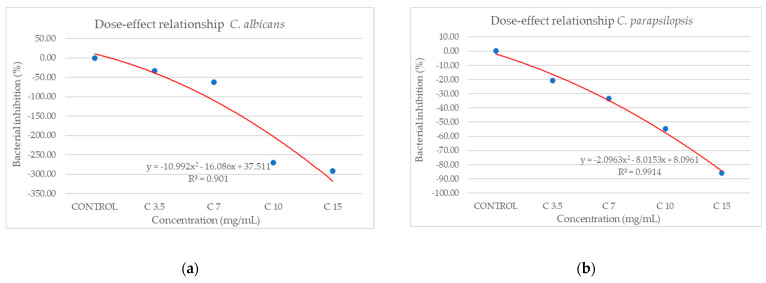
Dose–effect curves of the analyzed extract as a function of concentration (mg/mL) against (**a**) *C. albicans*, (**b**) *C. parapsilopsis*.

From the dose–effect curves (Figure 11 and Figure 12), it is observed that, except for the *S. flexneri* strain, increasing the concentration of the extract led to an increase in bacterial growth, following a polynomial relationship. Considering this trend in the case of *S. flexneri*, the possibility of evaluating the bactericidal/bacteriostatic effects in higher concentrations of extract should be investigated, in parallel with developed toxicological studies.

## 4. Discussion

In recent years, the activity of natural compounds derived from various medicinal plants has attracted considerable attention and has been extensively studied across multiple cancer pathologies. The novelty of the present study lies in the evaluation of the *C. libanotis* extract across several biological processes, including antioxidant, cell viability, cytotoxicity, and antimicrobial activity. Based on chromatographic and spectrometric data, furanocoumarins were confirmed as the predominant specialized metabolites found in the *C. libanotis* extract: xanthotoxin, bergapten, and isopimpinellin were the three dominant constituents. This extract also contained the pyranocoumarin seselin and the coumarin isogeijerin. Coumarins, especially bergapten, are natural compounds known for several pharmacological activities related to oxidation, such as photosensitizing activity, neuroprotection, organ protection, anticancer, anti-inflammatory, antimicrobial, and antidiabetic effects [21]. Bergapten’s potential has been acknowledged in various cancers, including liver, breast, lung, and colorectal cancer and melanoma. Its mechanisms of action involve apoptosis and the inhibition of cell proliferation [22]. Research into the anticancer activity of coumarin and its derivatives generally indicates that these compounds act through caspase-dependent apoptosis. According to the compositional profile of the hydroalcoholic extract developed for the present work from the aerial parts of *C. libanotis*, we evaluated its potential cytotoxic effects using a healthy human keratinocytes cell line (HaCaT) and a breast carcinoma cell line (MDA-MB-231). Our findings showed that human keratinocytes (HaCaT cells) did not appear to be negatively affected by the *C. libanotis* extract at concentrations ranging from 50 to 100 µg/mL. Furthermore, no significant changes in morphology were observed, with cells remaining adherent, maintaining confluence comparable to control cells, and preserving a typical epithelial morphology. These results suggest that the extract exhibits good biocompatibility with normal human cells at moderate concentrations, which is consistent with previous studies on coumarin-containing plant extracts. For instance, Gkionis et al. (2021) reported that low concentrations of natural furanocoumarins did not significantly affect the viability of normal human fibroblasts, supporting their potential for safe therapeutic use [23]. In contrast, the *C. libanotis* extract demonstrated a concentration-dependent cytotoxic effect on breast carcinoma cells (MDA-MB-231), significantly reducing cell viability and inducing pronounced morphological changes, including floating, rounded cells, loss of cell–cell adhesion, reduced confluence, and the accumulation of cellular debris. These findings align with earlier research showing that coumarins and furanocoumarins selectively target cancer cells. For example, Panno et al. (2009) demonstrated that bergapten and xanthotoxin, major furanocoumarins similar to those found in *C. libanotis*, induced apoptosis and inhibited proliferation in breast cancer cells by modulating key apoptotic and oxidative stress pathways [24]. Moreover, the morphological alterations observed in this study are characteristic of apoptosis-related cell death, as previously described in studies evaluating coumarin derivatives. Cui et al. (2019) reported that triphenylethylene-derived coumarins caused similar apoptotic features, including cell rounding and detachment in breast cancer cells, through the inhibition of angiogenesis-related signaling pathways [12]. It is important to note that although morphological alterations appeared more visually pronounced at 75 and 100 μg/mL, this was likely due to the greater number of adherent cells still present at those concentrations. At 125 and 150 μg/mL, the majority of affected cells were no longer adherent—having undergone extensive death and detachment—resulting in fewer visible morphological features despite higher cytotoxicity. This observation aligns with the MTT assay results, which showed a significant drop in viability at these higher concentrations. The selective cytotoxicity exhibited by the *C. libanotis* extract toward tumor cells while sparing normal keratinocytes highlights its therapeutic potential. This selectivity mirrors observations made with other medicinal plants rich in coumarins, such as *Ruta graveolens* and *Ammi majus*, where preferential cytotoxicity against cancer cells was documented. In a study by Fadlalla et al. (2011), extracts from *Ruta graveolens* exhibited strong antiproliferative effects on cancer cells based on the compounds that exert their effects by modulating specific molecular signaling pathways, thereby markedly suppressing the survival and proliferation of cancer cells [25]. While our findings support the cytotoxic activity of the *C. libanotis* extract against breast carcinoma cells and its relative safety toward normal keratinocytes, we acknowledge that the morphological data—particularly at the highest concentration tested (150 µg/mL)—should be interpreted with caution. Although viability assays indicate a substantial loss of live cells, morphological changes were less visually distinct in the representative images. We emphasize that this limitation is inherent to the qualitative nature of bright-field microscopy and have taken care not to overstate the conclusions. Future experiments will include quantitative morphological assessments and comparative testing with purified furanocoumarins to delineate the contribution of individual compounds.

Our study also demonstrated that the extract did not induce irritating effects on the chorioallantoic membrane and exhibited a good biocompatibility profile. No major alterations in the vascular plexus were observed following treatment with the extract. These findings are consistent with previous reports on plant-derived extracts rich in coumarins and related secondary metabolites. For example, furanocoumarins—a class of compounds commonly found in the Apiaceae and Rutaceae families—are known for their diverse biological activities, including antioxidant, anti-inflammatory, and cytoprotective effects, and they have been studied for their safety and pharmacological potential in various in vitro and in vivo models. These data suggest that coumarin- and furanocoumarin-rich extracts, when properly formulated, may offer safe therapeutic potential for topical or systemic applications [26,27]. Moreover, the low irritation score recorded for the *C. libanotis* extract suggests that coumarin-rich extracts, when properly formulated, may offer safe therapeutic potential for topical or systemic applications [28]. Our investigation of the antioxidant potential of *C. libanotis* extracts used a TBA assay, revealing a significant inhibitory effect on lipid peroxidation in bovine brain liposomes. These results are consistent with our previous research, in which *C. sicula* and *C. libanotis* extracts demonstrated a concentration-dependent radical scavenging ability using both a DPPH assay and a β-carotene bleaching assay. Specifically, three different extraction methods were used, and all the resulting extracts showed activity, with higher radical scavenging activity for alcoholic macerates than for samples obtained by pressurized cyclic solid–liquid extraction. Similarly, in the β-carotene bleaching method, the alcohol macerates maintained their superior antioxidant efficacy [5]. The observed inhibition of lipid peroxidation in the TBA assay, combined with the previously demonstrated radical scavenging and β-carotene bleaching activities, strongly supports the idea that *Cachrys* extracts possess significant antioxidant properties. This multifaceted antioxidant activity is most likely attributed to the presence of coumarins, known for their ability to neutralize free radicals and prevent oxidative damage [29]. Although other phytochemicals may also contribute, our ongoing research strongly suggests that coumarins play a significant role in the observed effects. The antimicrobial assessment of the *C. libanotis* extract produced varied results, demonstrating notable differences between Gram-positive and Gram-negative bacterial strains. Surprisingly, the majority of Gram-positive strains, such as *S. pyogenes*, *S. aureus*, *L. monocytogenes*, and *B. cereus*, exhibited a strain-boosting effect in a positive correlation to the increase in concentration. These results contradict the existing literature on the antibacterial properties of coumarin derivatives and require further investigation into the underlying mechanisms. The phytochemical composition of *C. libanotis* is primarily characterized by furanocoumarins, including xanthotoxin, bergapten, and isopimpinellin. Although these compounds exhibit cytotoxic and photosensitizing effects, their antimicrobial activity is more restricted and potentially selective. This might be possible since some bacterial species might be able to metabolize these compounds or evade their effects through efflux systems or enzymatic detoxification [30]. Furanocoumarins usually display antimicrobial effects by intercalating DNA and engaging photoreactive mechanisms, which necessitate UVA exposure for maximum effectiveness [31].

Studies on the antimicrobial activity of *C. libanotis* extract are few in the specialized literature. In their study, Aouachria et al. [32] examined the antimicrobial properties of different root extracts of *C. libanotis* from Alger. The extracts were prepared with solvents of increasing polarity, specifically hexane for defatting, chloroform for aglycone flavonoid extraction, and ethyl acetate for glycoside flavonoid extraction, against 11 selected human pathogens bacterial strains: Gram− bacteria (*Pseudomonas aeruginosa*, *E. coli*, *S. typhimurium*, *A. baumanii*, *C. freundii*, *P. mirabilis*, *K. pneumoniae*) and Gram+ bacteria (*S. aureus*, *B. cereus*, *E. faecalis*, *L. monocytogenes*) using the agar disc diffusion method. All extracts exhibited lower inhibitory potential than Gentamicin across all tested strains. In contrast to our findings, their study indicated that Gram-positive bacteria were more susceptible to the *C. libanotis* extract than Gram-negative bacteria, which proved more resistant to the treatment. This differentiated behavior may be due to the specificity of the existing chemotypes in the composition of the extracts, the extraction solvent, the agroclimatic, pedological conditions, and the methods of sampling and storage particular to each region [33].

Given our results obtained regarding the antimicrobial activity of *C. libanotis* ethanolic extract, we can conclude that future activity can focus on studies regarding the inhibition of two Gram-negative pathogens *Shigella flexneri* and *Salmonella typhimurium* for which the inhibition rates are promising. The present findings highlight the intricate nature of phytocomplex–microbe interactions and emphasize the importance of improving antimicrobial evaluations based on both chemotype and microbial target. They further indicate that although *C. libanotis* extract exhibits restricted broad-spectrum antibacterial properties, it may possess selective inhibitory capabilities, especially against Gram-negative enteric pathogens. *S. flexneri* is a Gram-negative bacterium endemic in developing countries that causes most bacterial dysenteries, shigellosis, and more mortality than any other *Shigella* species [34]. *S. flexneri* is increasingly developing antibiotic resistance. *S. typhimurium* is a pathogen that most often causes gastroenteritis in humans. Infection is possible from ingestion of infected prey, infected food sources, or from a contaminated environment, including hospitals. *S. typhimurium* is usually resistant to ampicillin, chloramphenicol, streptomycin, sulfonamides, and tetracycline [33]. Under these conditions, in developing countries, finding alternative plant-based antibiotics is a viable solution from an economic point of view. In this sense, proof of the effectiveness of the extract of *C. libanotis* against *S. flexneri* and *S. typhimurium*, found in important quantities in spontaneous flora, represents an alternative to the expensive, strong synthetic antibiotics.

## 5. Conclusions

Data on the activity of several natural compounds obtained from various medicinal plants have captured attention in recent years and are widely studied and tested in various cancer pathologies. What is new in this study is the assessment of the *C. libanotis* sample’s influence on various biological processes, including cellular health, toxicity to cells, and microorganism proliferation. Regarding antimicrobial activity, the extract under review produced variable results, with varying effects on Gram-positive and Gram-negative bacterial strains, suggesting potential selectivity and the need for further investigation of mechanisms of action and interactions with specific Gram-negative pathogens such as *S. flexneri* and *S. typhimurium*. The *C. libanotis* extract selectively reduced breast carcinoma cell viability while preserving the integrity and morphology of healthy keratinocytes. Morphological changes confirmed apoptotic features in cancer cells, and the low irritation score observed in the HET-CAM assay highlighted its excellent biocompatibility. These findings position *C. libanotis* as a promising candidate for the development of safe, plant-based anticancer therapies. While the findings of this study provide promising initial evidence of the selective cytotoxic potential of *C. libanotis* extract (Cex) against MDA-MB-231 breast cancer cells, several limitations should be acknowledged. First, only one cancer cell line (MDA-MB-231) and one normal cell line (HaCaT) were used, which limits the generalizability of the observed selectivity. Additional cancer models representing different subtypes and tissues, as well as in vivo validation, are necessary to confirm the broader anticancer potential of the extract. Second, although the scratch assay suggested a reduction in cell migration, the interpretation is complicated by cytotoxic effects observed at higher concentrations, as evidenced by significant morphological alterations and viability loss at 48 h. Therefore, the possibility that reduced migration is partially due to cell death rather than specific inhibition of migratory capacity cannot be excluded. Third, while the differential response between cancerous and non-cancerous cells is notable, the present study did not explore the underlying molecular mechanisms responsible for this selectivity. These limitations have been clearly acknowledged and discussed to provide appropriate context for the interpretation of our results and to guide future research directions.

## Figures and Tables

**Table 1:** Irritation scores obtained for the *C. libanotis* extract and the two controls.

IS	H_2_O	1%SDS	*C. libanotis* Extract 100 µg/mL
IS	0.14	19.41	0.78
tH	300	19 s	300
tL	297	23 s	295
tC	300	27 s	280

## Data Availability

Data is contained within the article or Supplementary Material.

## Supplementary Materials

The following supporting information can be downloaded at: https://www.mdpi.com/article/10.3390/antiox14070810/s1, Figure S1: Compounds were identified according to their retention times and mass spectra.
