# Supplementary material for: Pharmaco-Toxicological Effects of Cachrys libanotis Extract: Antioxidant, Antimicrobial, and Cytotoxic Activities in Human Cell Lines and Embryonic Models
Source: Antioxidants (Basel). 2025 Jun 30;14(7):810. doi: 10.3390/antiox14070810 (PMC12291932; doi:10.3390/antiox14070810)
Supplement: Supplementary file 1 [file antioxidants-14-00810-s001.zip › antioxidants-3647735-supplementary.pdf]

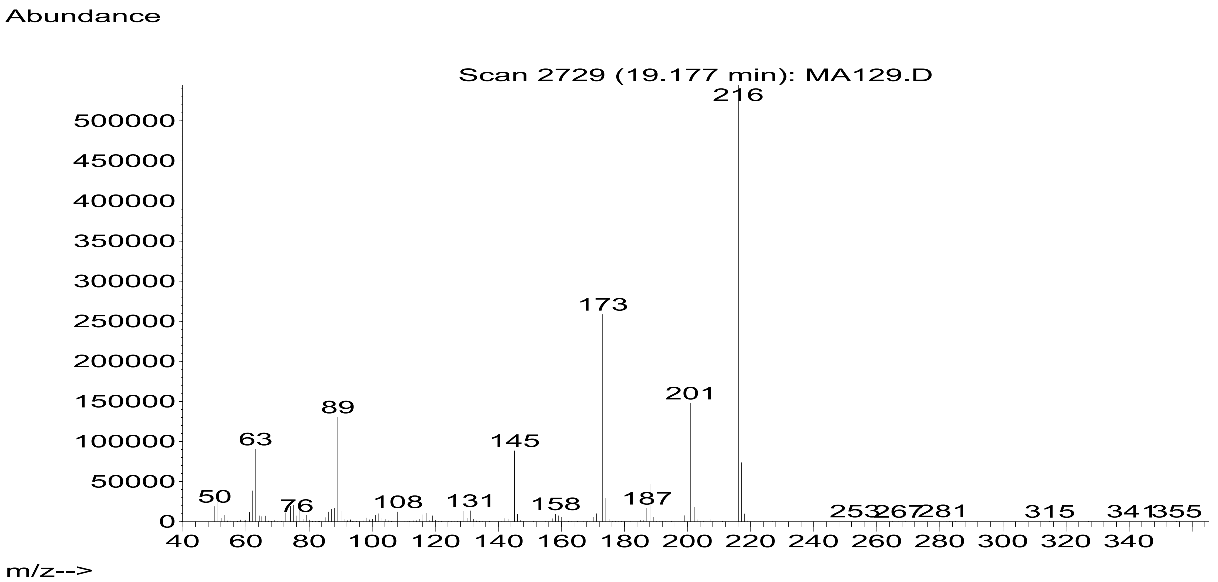

Xantotoxin

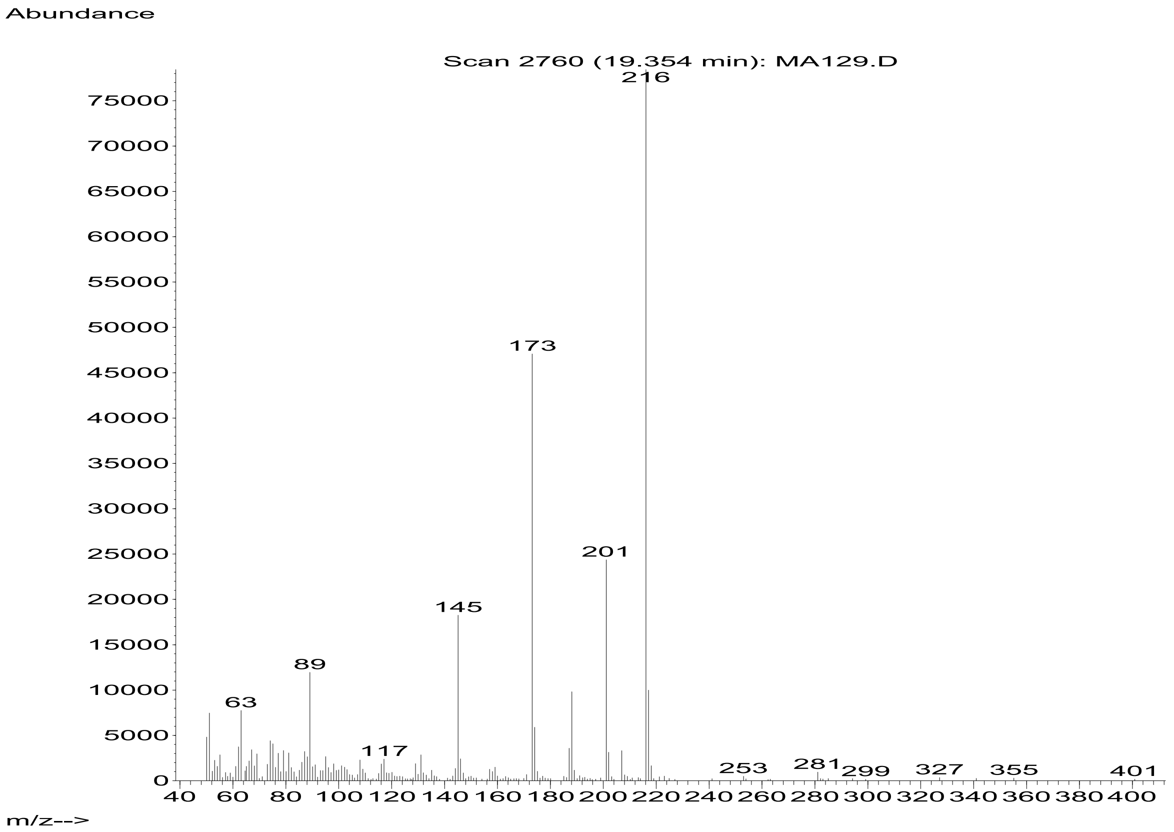

Bergapten

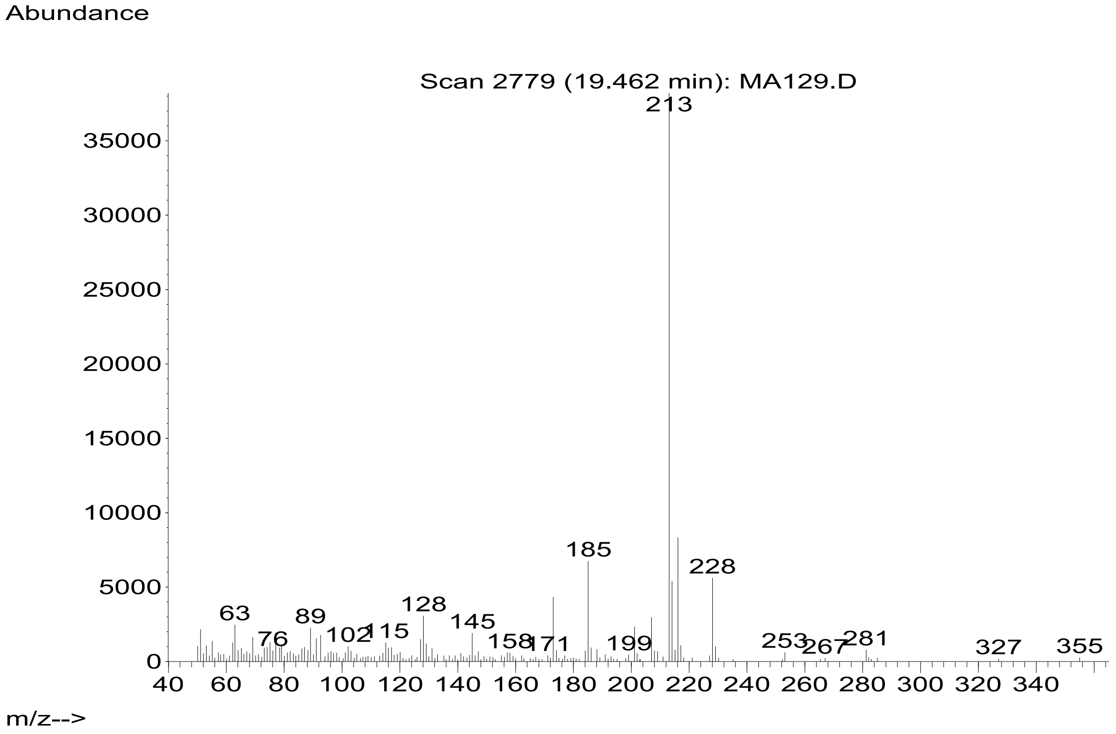

Seselin

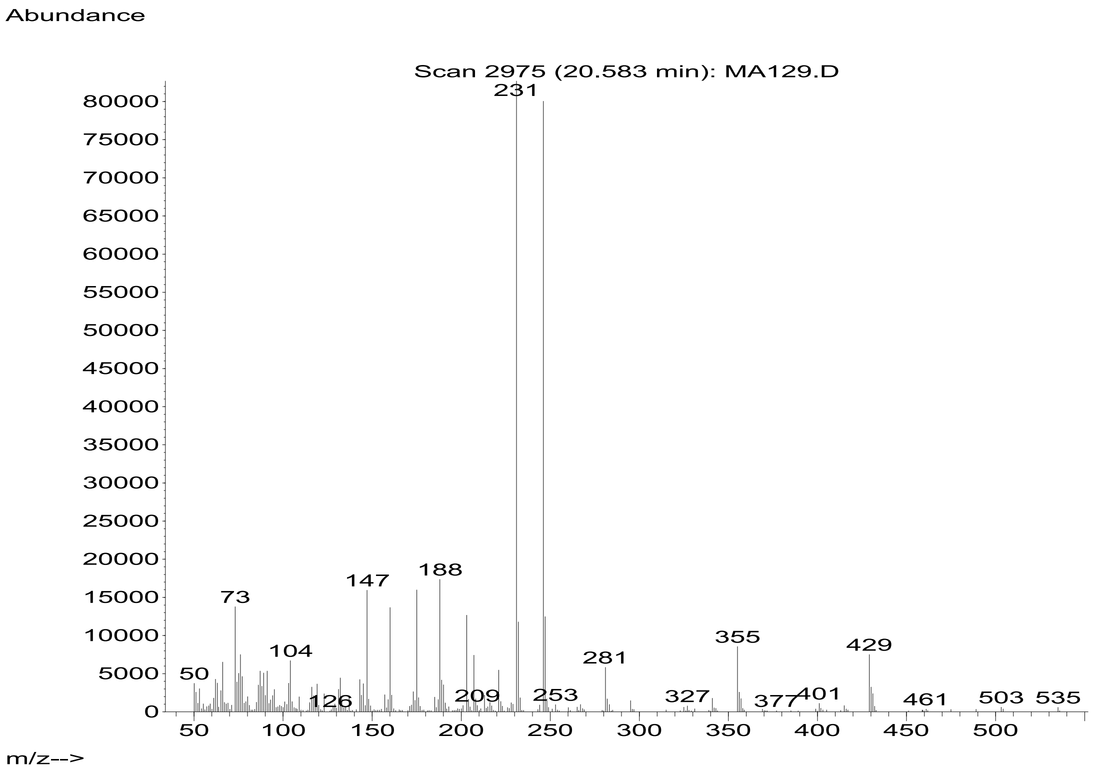

Isopimpinelin

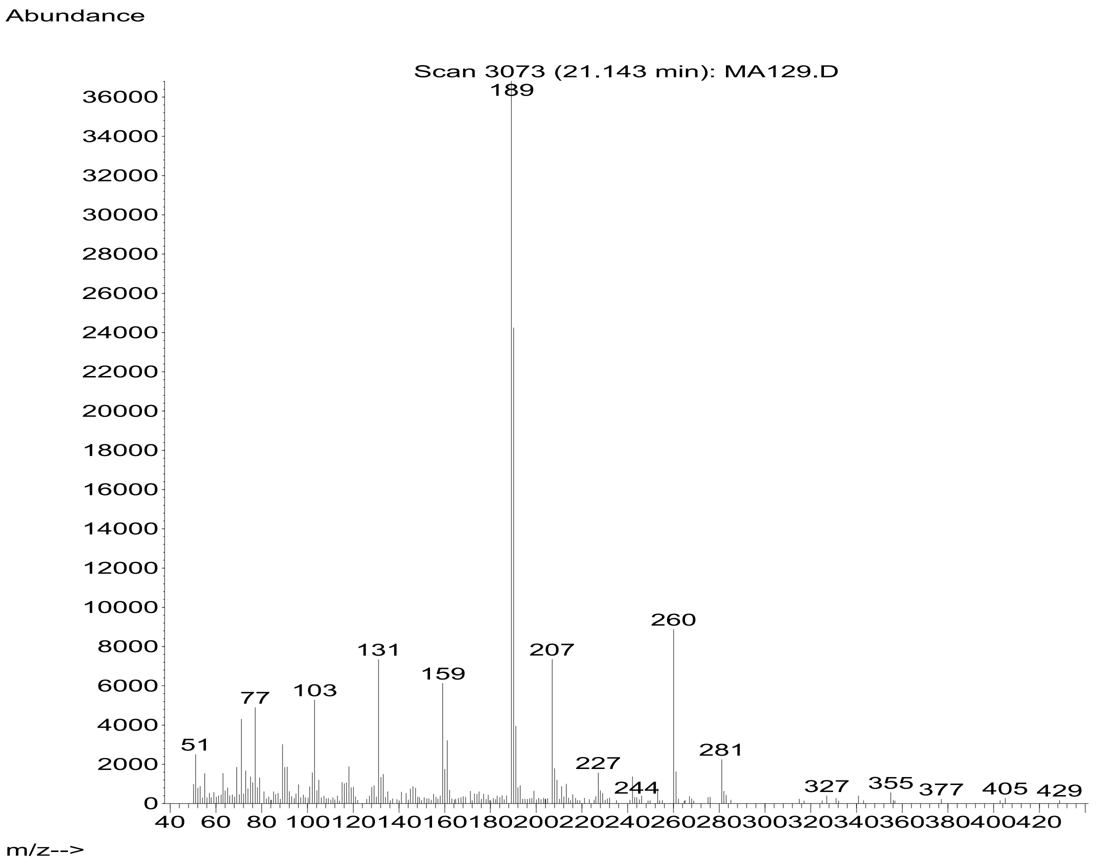

Isogeijerin
